# Intérêt de la scintigraphie lymphatique et place de la chirurgie dans les chylothorax congénitaux

**DOI:** 10.11604/pamj.2016.24.321.8014

**Published:** 2016-08-22

**Authors:** Jihène Methlouthi, Nabiha Mahdhaoui, Manel Bellalah, Nouir Selsabil, Ayache Hedia, Raja Sfar, Habib Essabah, Nouri Abdellatif, Nouri Sonia, Seboui Hassen

**Affiliations:** 1 Faculté de Médecine de Sousse, Unité de Réanimation Néonatale Code (UR/99/UR/08-65), Service de Néonatologie CHU Farhat Hached Sousse, Tunisie; 2 Service de Médecine Nucléaire CHU Sahloul Sousse, Tunisie; 3 Service de Chirurgie Pédiatrique CHU F Bourguiba Monastir, Tunisie

**Keywords:** Nouveau-né, chylothorax congénital, scintigraphie lymphatique, chirurgie, New-born baby, congenital chylothorax, lymphatic scintigraphy, surgery

## Abstract

Le chylothorax est défini par l'accumulation du liquide lymphatique dans la cavité pleurale. Il existe 3 catégories distinctes chez le nouveau-né: les chylothorax congénitaux (CC), les chylothorax malformatifs ou syndromiques (CM) et les chylothorax post-opératoires (CO). Bien que rare, le chylothorax représente la cause la plus fréquente des épanchements pleuraux en période néonatale. Son diagnostic positif est facile par analyse du liquide pleural, mais son mécanisme et surtout l'intégrité du canal thoracique et ses collatérales est parfois difficile à préciser. La lymphoscintigraphie représente l'examen de choix dans le diagnostic etipathogénique. Ce moyen de diagnostic peut être couplé, si possible, au SPECT-CT (single photon emission tomography/computed tomography) permettant de donner des renseignements plus précis, notamment sur le plan anatomique. Sa prise en charge repose sur le drainage du liquide pleural, la suppression des graisses alimentaires et la nutrition parentérale. Le recours à la chirurgie est préconisé devant l'échec du traitement médical. Nous rapportons l'observation d'un nouveau-né porteur d'un chylothorax unilatéral, n'ayant pas répondu au traitement médical. La lymphoscintigraphie avait permis de diagnostiquer le mécanisme étiopathogénique et par conséquent, de guider le traitement chirurgical.

## Abstract (en)

Chylothorax is defined as accumulation of lymphatic fluid in the pleural space. Chylothorax in the neonatal period can be classified into three distinct categories: congenital chylothorax (CC), syndromic or malformative chylothorax (MC) and postoperative chylothorax (CO). Although rare, chylothorax is the most common cause of pleural effusions in the neonatal period. Making a positive diagnosis is easy by examination of the pleural fluid, but its mechanism and especially the integrity of thoracic duct and its collateral branches is sometimes difficult to determine. Lymphoscintigraphy is the test of choice in etipathogenic diagnosis. This diagnostic tool can be coupled, if possible, to SPECT-CT (single photon emission tomography / computed tomography) providing more specific anatomical informations. Tratment of chylothorax is based on the drainage of the pleural fluid, the suppression of dietary fats and on parenteral nutrition. Surgery is recommended in the case of medical treatment failure. We report the case of a newborn with unilateral chylothorax who did not respond to medical treatment. Lymphoscintigraphy allowed to diagnose etiopathogenic mechanism underlying chylothorax and therefore to direct surgical treatment.

## Introduction

Le chylothorax est défini par l'accumulation du liquide lymphatique dans la cavité pleurale [1]. Il existe 3 catégories distinctes chez le nouveau-né: les chylothorax congénitaux (CC), les chylothorax malformatifs ou syndromiques (CM) et les chylothorax post-opératoires (CO) [2]. Son diagnostic positif est facile par la simple ponction pleurale [3], mais son mécanisme et surtout l'intégrité du canal thoracique et ses collatéraux est parfois difficile à préciser. Son traitement est habituellement médical et la chirurgie reste le dernier recours [1]. Nous rapportons l'observation d'un nouveau-né porteur d'un chylothorax unilatéral n'ayant pas répondu au traitement médical avec une évolution favorable après un traitement chirurgical basé sur les données de la lympho-scintigraphie. A travers ce cas, nous essayerons d'établir l'intérêt de la scintigraphie lymphatique et la place de la chirurgie dans le traitement du chylothorax congénital.

## Patient et observation

**Figure 1:**
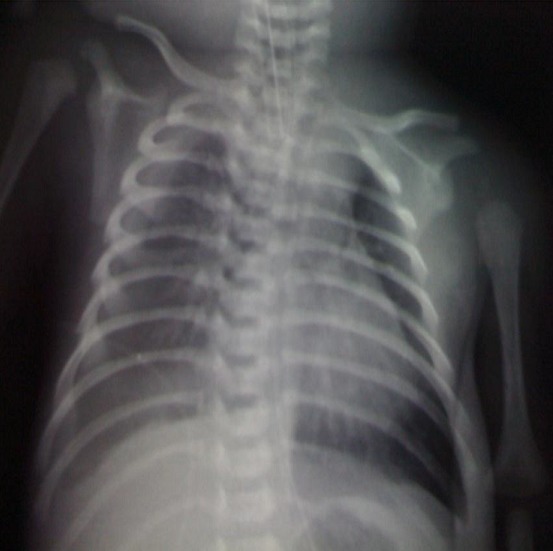
Radiographie du thorax montrant un épanchement pleural droit de grande abundance

**Figure 2:**
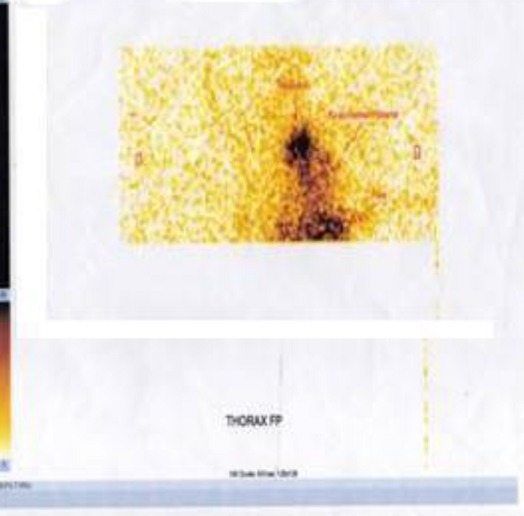
Scintigraphie lymphatique: fixation du radiotraceur en projection de la base du thorax droit

Nouveau-né de sexe masculin, né à terme d'une mère âgée de 25 ans, primipare primigeste, sans antécédents particuliers. La grossesse était de déroulement normal et l'accouchement était par voie basse sans incidents avec une bonne adaptation à la vie extra-utérine. Le nouveau-né était eutrophique avec un poids de naissance de 2910g, une taille de 49cm et un périmètre crânien de 34 cm. Il avait une dysmorphie faciale rappelant la trisomie 21 et une détresse respiratoire immédiate ayant nécessité son transfert dans l'unité de réanimation néonatale. L'examen avait noté, par ailleurs, une saturation en oxygène à l'air ambiant à 90%, un état hémodynamique stable, une auscultation cardiaque sans anomalies et une auscultation pulmonaire asymétrique avec une nette diminution des murmures vésiculaires du côté droit. La radiographie du thorax avait montré un hémithorax droit opaque avec un cœur refoulé à gauche faisant suspecter un épanchement pleural liquidien (Figure 1). L'échographie thoracique a confirmé ce diagnostic. C'était un épanchement de grande abondance dont la ponction avait ramené 50 ml de liquide jaune citrin. L'analyse cytochimique avait conclu à un chylothorax avec une pleïocytose à 1500 éléments/mm^3^ à prédominance lymphocytaitre et une hypertriglycéridémie. L'évolution ultérieure était marquée par la récidive de l'épanchement pleural et la dégradation de l'état respiratoire du nouveau-né nécessitant le recours à la ventilation assistée pendant 48 heures et la mise en place d'un drain pleural droit. Il a été alimenté par voie parentérale exclusive pendant 20 jours, relayée par une alimentation orale à base de dérivé lacté riche en triglycérides à chaînes moyennes. Devant l'absence de tarissement de l'épanchement pleural et afin de comprendre le mécanisme de ce chylothorax, une scintigraphie lymphatique a été pratiquée à J32 de vie. Elle avait mis en évidence une fixation du radiotraceur en projection de la base du thorax droit confirmant l'extravasation du liquide lymphatique dans la cavité pleurale droite (Figure 2). Le nouveau-né a été opéré à J59 de vie à travers une thoracotomie postéro latérale droite. Les chirurgiens ont pu mettre en évidence, à la face postérieure de l'aorte la présence d'un vaisseau lymphatique faisant 1mm de diamètre et qui éjecte de la lymphe à 15 mm du diaphragme. L'écoulement s'est arrêté après ligature de ce vaisseau. L'évolution était favorable avec absence de récidive de l'épanchement pleural même après la reprise d'une alimentation lactée normale. Par ailleurs, le caryotype avait conclu à une trisomie 21 libre et homogène. Un bilan morphologique comportant une échographie cardiaque, rénale, trans-fontanellaire ainsi qu'une radiographie du squelette était sans anomalies. En revanche, il avait une hypothyroïdie périphérique. Le nouveau-né était mis sortant à J110 de vie, il est actuellement âgé de 5 ans, toujours suivi à la consultation externe avec une évolution favorable.

## Discussion

Bien que rare, le chylothorax représente la cause la plus fréquente des épanchements pleuraux en période néonatale. Son incidence étant estimée à 1/15000 naissances vivantes [1, 4]. L'étiologie des chylothorax congénitaux est en général inconnue. Des anomalies du système lymphatique thoracique sont incriminées, telle qu'une malformation, une absence ou une atrésie du canal thoracique, ou encore une fistule congénitale pleurolymphatique par défaut de connexion lymphatique. Ces anomalies lymphatiques localisées peuvent s'associer à des malformations plus diffuses (lymphangiectasie intestinale, lymphangiome kystique, lymphoedème cutané), ou s'intégrer dans des syndromes connus tels le syndromes de Noonan, la maladie de Turner, les maladies lysosomiales ou encore la trisomie 21 comme c'était le cas pour notre patient [5–7]. D'autres mécanismes étiopathogéniques sont plus récemment établis tel qu'une rupture du canal thoracique ou de l'un de ses affluents ou collatérales, siège d'un reflux de chyle à partir du canal thoracique du fait de la perte malformative de sa continence valvulaire. Dans ces cas, la rupture pourrait être en rapport avec un à-coup d'hyperpression dans ce territoire de lymphangiectasie distendu par le reflux [8, 9]. Dans les autres chylothorax congénitaux et plus particulièrement en cas de malformation du canal thoracique et/ou de son origine, la lymphe contenant le chyle est obligée d'emprunter les autres voies et courants lymphatiques thoraciques pour rejoindre les confluents veineux du cou. Le développement de la circulation dans ces voies va gêner l'évacuation des lymphatiques intercostales qui se distendent et perdent leur jeu valvulaire. Le chyle va alors refluer et rejoindre la circulation veineuse, soit par les voies mammaires ou axillaires externes, soit en refluant encore plus en avant vers les voies thoraciques antérieures (mammaires internes). À noter que le diaphragme, de part sa richesse lymphatique, peut jouer un rôle actif important dans la dérivation du chyle. Le chyle intestinal, qui ne peut rejoindre l'origine du canal thoracique, peut également emprunter les lymphatiques hépatiques et rejoindre les lymphatiques du diaphragme par l'intermédiaire du ligament suspenseur du foie. Le chyle rejoint alors les veines du cou par la voie lymphatique pariétale antérieure mais aussi par les courants médiastinaux. À partir des chaînes ganglionnaires péritrachéobronchiques, le chyle et la lymphe peuvent, par le même mécanisme de perte de jeu valvulaire, refluer dans les vaisseaux lymphatiques intrapulmonaires jusqu'à engorger les vaisseaux lymphatiques de la plèvre viscérale. Les chylothorax peuvent survenir à partir de la rupture des vaisseaux surdistendus pouvant siéger: au niveau de la plèvre pariétale et/ou médiastinale, du diaphragme, de la plèvre viscérale. Ces ruptures peuvent être uniques ou multiples, facilement visibles ou minuscules [8]. Le diagnostic du chylothorax est établi par l'analyse du liquide pleural qui montre un taux de triglycérides supérieur ou égal à 1,1 mmol/l, une cellularité > 1000 ou une lymphocytose supérieure à 80% [1, 7]. Une mise en évidence du canal thoracique et de ses états pathologiques est primordiale afin de guider le traitement. La lymphographie pédieuse était l'examen clé dans l'étude de l'étiopathogénie des chylothorax. Elle peut montrer l'absence du canal thoracique et parfois les voies de dérivation du chyle qui le suppléent. Le canal thoracique peut être perméable et un reflux dans un affluent incontinent peut être objectivé. Parfois on ne retrouve que les signes indirects de cette perméabilité. L'embolisation du canal thoracique par lymphographie reste l'une de ses rares indications d'où son intérêt en préopératoire couplée au scanner pour situer précisément le siège lésionnel [10, 11]. Actuellement, cet examen tend à être largement remplacé par des lympho-scintigraphies au TC99m-HAS (Human serum albumin) dont le principe et la pratique sont simples. Des particules colloïdales de petite taille radio-marquées sont injectées dans le tissu étudié, sont drainées par les terminaisons lymphatiques et par conséquent transportées dans les vaisseaux lymphatiques [11]. C'est un moyen qui permet de nous renseigner sur les anomalies anatomiques et fonctionnelles des voies lymphatiques. Ce moyen de diagnostic lorsqu'il est couplé au SPECT-CT (single photon emission tomography / computed tomography) permet de donner des renseignements plus précis, notamment sur le plan anatomique. En effet, cette technique vient compléter les images classiques de l'ensemble du corps en fournissant des images scintigraphiques et vues en coupes CT dans tous les plans de l'espace [9, 12–14]. Le traitement du chylothorax est essentiellement médical [1, 3, 15], visant d'une part à assurer la vacuité pleurale à l'aide de ponctions pleurales ou drainage en cas de récidive [8] et d'autre part à diminuer la production du chyle par une alimentation parentérale totale d'une durée variable relayée par un dérivé lacté riche en triglycérides à chaînes moyennes qui, déversés directement dans le système porte, shuntent le système lymphatique. Certains médicaments comme la somatostatine ou son analogue l'octréotide réduisent les sécrétions digestives, la pression veineuse intrahépatique, le débit sanguin splanchnique et par conséquent la production du chyle [3, 11]. A coté de ces différents moyens il faut rétablir et/ou maintenir l'état nutritionnel et l'homéostasie avec un équilibre protidique et électrolytique [15, 16]. Exceptionnellement, le traitement est chirurgical de dernier recours, guidé par la lymphoscintigraphie qui va permettre d'identifier le mécanisme exact du chylothorax. Cette chirurgie consiste le plus souvent à ligaturer le canal thoracique ou à suturer les fuites au niveau des collatérales [2, 8]. Dans les cas difficiles et quand l'origine du chyle n'est pas identifiable, on se contente d'une symphyse pleurale [15, 16].

## Conclusion

Le traitement du chylothorax congénital est dans la quasi-totalité des cas médical. Néanmoins, il est important d'établir des éléments pronostiques autorisant le recours à la chirurgie de façon plus précoce et pertinente. D'où l'importance d'une bonne compréhension du mécanisme physiopathologique et par conséquent l'importance d'une exploration par lymphographie ou lympho-scintigraphie dans les chylothorax résistant au traitement médical.
